# Cardioprotective effects of Schisantherin A against isoproterenol-induced acute myocardial infarction through amelioration of oxidative stress and inflammation via modulation of PI3K-AKT/Nrf2/ARE and TLR4/MAPK/NF-κB pathways in rats

**DOI:** 10.1186/s12906-023-04081-x

**Published:** 2023-08-04

**Authors:** Xiaolong Mi, Zhijun Zhang, Jinfang Cheng, Zheng Xu, Kaiyi Zhu, Yunxia Ren

**Affiliations:** 1 Department of Cardiovascular Medicine, Shanxi Bethune Hospital, Shanxi Academy of Medical Sciences, Tongji Shanxi Hospital, Third Hospital of Shanxi Medical University, Taiyuan, 030032 China; 2 Department of Cardiovascular Medicine, Tongji Hospital, Tongji Medical College, Huazhong University of Science and Technology, Wuhan, 430030 China

**Keywords:** Cardioprotection, Schisantherin A, Myocardial infarction, Oxidative stress, Inflammation

## Abstract

**Background and aims:**

The scientific community is concerned about cardiovascular disease mortality and morbidity, especially myocardial infarction (MI). Schisantherin A (SCA), a dibenzocyclooctadiene lignan monomer found in *S. chinensis* fruits has cardiovascular advantages such as increasing NO production in isolated rat thoracic aorta and reducing heart damage caused by ischemia-reperfusion (I/R) through decreasing apoptosis. The present study was undertaken to explore the potential effects of SCA on ISO-induced myocardial infarction in rats.

**Methods:**

Rats were randomly allocated to four groups: control; ISO-treated, and two additional groups of ISO + SCA (5 or 10 mg/kg body weight). All SCA-treated groups were administered with SCA for 20 days and all ISO groups were challenged with ISO on days 19 and 20.

**Results:**

SCA significantly attenuated ISO-induced rise in heart/body weight ratio, myocardial infarct size, and cardiac functional biomarkers (CK-MB, cTnI and BNP). SCA pre- and co-treatment resulted in a significant reduction in oxidative stress (via MDA, NO and GSH and increased activities of SOD, CAT and GPx) and inflammation (via decreased levels of TNF-α, IL-6 and IL-1β) markers when compared to the same levels in cardiac tissue of ISO-treated rats. This study also showed that SCA protects ISO-induced oxidative stress and inflammation by activating the PI3K-AKT/Nrf2/ARE pathway and suppressing TLR4/MAPK/NF-κB pathways. Furthermore, SCA treatment protected histopathological alterations observed in only ISO-treated cardiac transverse sections of rats.

**Conclusion:**

In conclusion, the findings of this study suggest that SCA protects against cardiac injury in the ISO-induced MI model of rats.

## Introduction

Cardiovascular diseases (CVDs) are a group of heart and blood vessel disorders that are responsible for the vast majority of deaths worldwide. In 2019, CVDs were responsible for an estimated 17.9 million deaths, representing approximately 32% of all global deaths [1]. Heart attacks and strokes accounted for 85% of these deaths [1]. According to recent data from the World Health Organization (WHO), CVDs were attributed to nearly 40% of the 17 million premature deaths caused by noncommunicable diseases in 2019 [1]. Furthermore, it is estimated that CVD contributes to approximately 18.6 million deaths annually [1]. A myocardial infarction (MI), also known as a heart attack, occurs when blood flow to the heart muscle, or myocardium, is reduced or completely stopped [2]. The heart undergoes extensive myocardial remodeling post-MI, affecting cardiac function [3, 4]. A large body of scientific evidence has demonstrated that loss of oxidative homeostasis in myocardial cells results in excessive generation of cytotoxic free radicals in MI conditions [5, 6]. Previously, NJ Pluijmert, CI Bart, WH Bax, PH Quax and DE Atsma [4] stated that myocardial cells in oxygen-deprived conditions experienced an inflammatory response by instigating inflammatory molecules. Furthermore, a plethora of non-clinical scientific evidence has demonstrated that inflammation is initiated through the activation of mitogen-activated protein kinases (MAPK) in association with Nuclear factor kappa-light-chain-enhancer of activated B cells (NF-kB) and subsequent production of high levels of pro-inflammatory mediators including cytokines [7], making inflammatory response a potential target for therapeutic interventions.

Although treatment options such as Angiotensin-Converting Enzyme (ACE) inhibitors and beta-blockers are available, these are not suitable for patients with blood pressure below 90/60mmhg and chronic respiratory diseases (recurrent cough and dyspnea). Due to the limitations of available drugs, there is an urgent need to develop alternative therapeutic options to prevent myocardial remodeling post-MI and progression to heart failure. Historically, natural products have been part and parcel of drug discovery and development and a rich source of drugs to treat various diseases including CVD [8].

*Schisandra chinensis* (Turcz.) Baill. *(S. chinensis)* is a Chinese traditional medicine listed as an herbal supplement in western phytotherapy. Its bioactive components have been used as adaptogens in alternative medicine to treat various ailments, including cardiovascular disease [9]. Schisantherin A (SCA), also known as gomisin C or wu-wei-zi ester A, a dibenzocyclooctadiene lignan monomer is isolated from the fruits of *S. chinensis* [10]. It has been considered a primary biologically active lignan and has been opted as one of the reference standards for quality checks of *S. chinensis* end products in Chinese Pharmacopeia [11]. SCA is well-known for its health-promoting effects showing diversified beneficial pharmacological properties such as anti-cancer [12], anti-oxidative [13, 14], anti-inflammatory in vitro and in vivo [15]. Earlier, it was also proved that SCA could protect the liver [16] or kidney [17] against ischemia-reperfusion (I/R) injury, Parkinson’s disease [18], improves learning and memory [14].

*S. chinensis* has been utilized in traditional eastern medicine to treat various cardiovascular conditions, and recent research has shown its efficacy in treating hypertension and myocardial infarction [19]. Superoxide scavenging, NADPH oxidase inhibition, eNOS phosphorylation, actomyosin activation signal interference, and NADPH oxidase inhibition are some of the mechanisms by which SCA and its active chemicals increase vasodilation [20]. *In-vitro* signaling pathway aberrations in SCA include the death of human gastric cancer cells by changes in the Reactive oxygen species/ Jun N-terminal kinase (ROS/JNK) signaling system [21], reduce interleukin-1β-induced inflammation in human chondrocytes by decreasing NF-κB and MAPK activation [22]. whereas the PI3K/Akt signaling pathway protects renal tubular epithelial cells from hypoxia/reoxygenation damage [23]. *In-vivo* studies have revealed that SCA effectively inhibits NF-κB and MAPK activation, resulting in strong anti-inflammatory effects in an lipopolysaccharide (LPS)-exposed mice [24]. SCA has also been shown to increase antioxidant capacity in rats, potentially relieving D-galactose-induced learning and memory impairments [25]. SCA exerted vasorelaxant effects on an isolated rat thoracic aorta, which may be attributed to its stimulation of nitric oxide (NO) and prostacyclin (PGI2) synthesis, as well as its suppression of voltage-dependent calcium channel (VDCC) activation [26]. SCA may reduce cardiac damage brought on by ischemia-reperfusion (I/R) by lowering apoptosis, according to an *in-vitro* and *in-vivo* study [27]. The possibility that SCA might reduce I/R damage by activating the Toll-like receptor 4 (TLR4) and Complement component 5a receptor 1 (C5aR1) signaling pathways has also been studied [28].

Despite these promising results, SCA must establish that it is beneficial and safe to use in animal models before it can be used on people. To address this gap, a study was conducted to investigate the preventative effects of SCA on acute myocardial infarction in rats using an ISO-induced myocardial infarction model. The study looked at the potential significance of multiple signaling pathways and various metrics related to cardiac function, myocardial infarction, oxidative and inflammatory markers. More research is required to properly grasp the potential of SCE and its components as treatments for cardiovascular diseases.

## Materials and methods

### Drugs and chemicals

Schisantherin A (SCA) and isoproterenol (ISO) were supplied by Sigma Chemical Company in St. Louis, Missouri. All other chemicals used in the study were of analytical grade and purchased from reputable chemical suppliers.

### Animals

Male Wistar albino rats (8–10 weeks) were procured from an authorized animal vendor. After procuring animals, veterinarians assessed their health condition, and healthy animals were selected for the study. Animals were acclimatized for a week before the initiation of treatment. During the acclimatization period, veterinarians used a marker pen to identify individual animals by temporary numbers on the tail and observed them for ophthalmological and detailed health examinations. Animals were housed in a well-controlled environment of the experimental animal room with a temperature of 19–25 °C, relative humidity of 30–70%, a light/dark cycle of ~ 12 h and 15–20 fresh air changes per hour. Animals were housed in a maximum of 4 per cage throughout the study period in sterilized polypropylene (Dimension; L 410 x B 290 x H 175 mm) rodent cages covered with stainless steel grid top mesh having provision for water bottles and feed (free of microbes and contaminants; adequate nutritional components). Autoclaved clean corn cob (free of microbes and contaminants) was used as a bedding material.

### Experimental design

#### Induction of myocardial infarction (MI) in rats

**Fig. 1:**
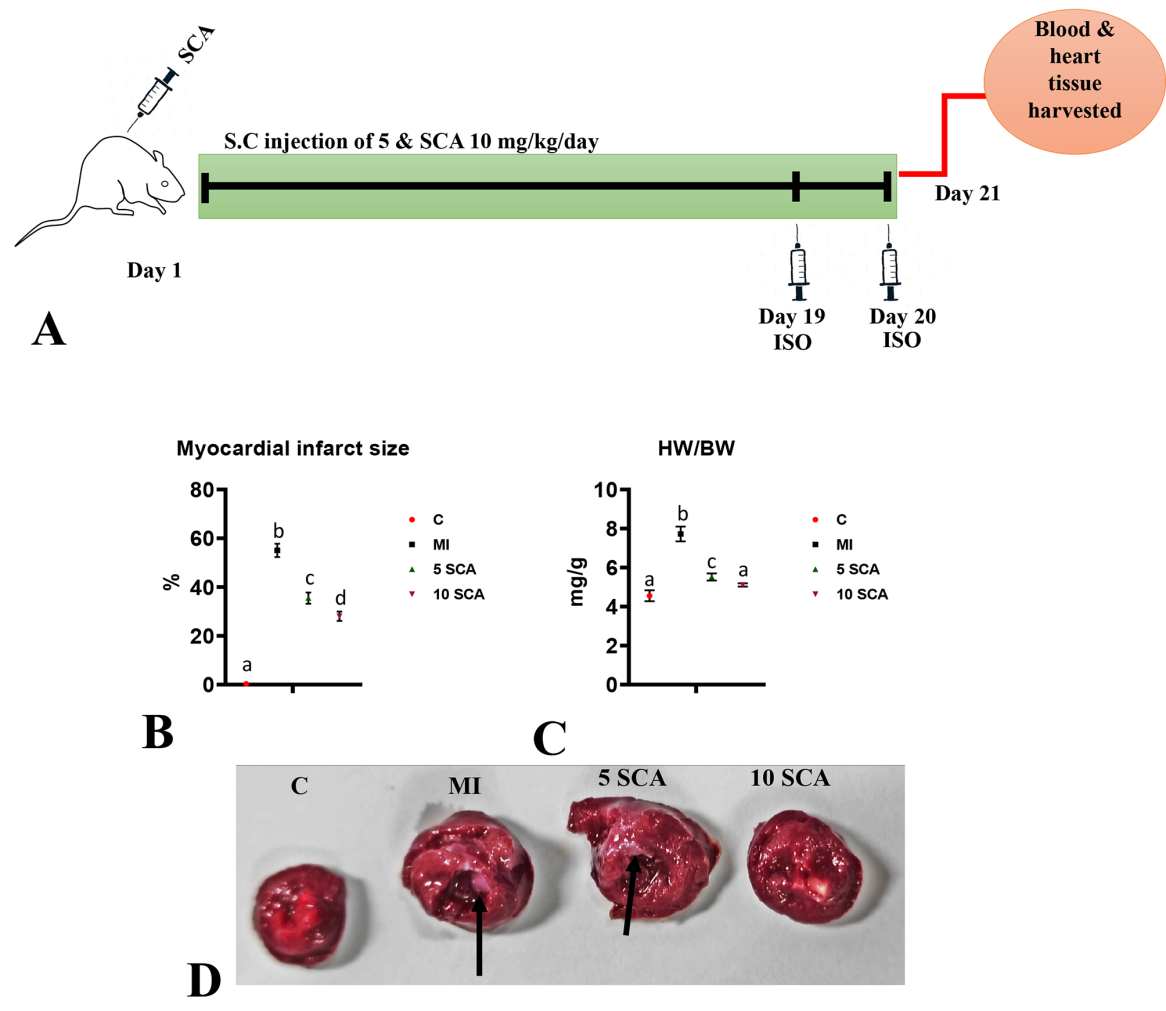
**(A)**: Schematic diagram of experimental design. Effect of SCA treatment on **(B)**: Myocardial infarct size **(C)**: Heart-to-body weight ratio in ISO-injected rats. **(D)**: The myocardial infarct size was assessed through a histological TTC staining assay. The myocardial infarct area is indicated by the black arrow. C: Control rats; MI: Isoproterenol (ISO) induced myocardial infarction (MI) in rats; 5 SCA: Treatment of ISO-induced rats with 5 mg SCA/ kg body weight; 10 SCA: Treatment of ISO-induced rats with 10 mg SCA/ kg body weight. The data is presented as mean ± SD (n = 10). Statistical analysis was performed using One-Way ANOVA followed by Tukey’s post hoc analysis to compare differences between groups. Mean values with different superscripts indicate significant differences at a significance level of p < 0.05. S.C: Subcutaneous; ISO: isoproterenol

Animals were randomized into treatment and control groups by a zig-zag method based on body weights using Microsoft excel to allocate 10 animals per group in G1 to G4 groups. The body weight variation did not exceed ± 20% of the mean body weight range. Each cage was identified by a color-coded label with details such as Cage No., Dose, Group, Species, Strain, Sex, Animal ID, Treatment Start date, and Date of Necropsy. Choosing an animal model to mimic human myocardial infarction (MI) is vital for research. Isoproterenol (ISO), a non-selective β-agonist, is commonly employed to induce MI in animal studies. This model is widely accepted for its simplicity, reproducibility, and lower mortality rate compared to other models [29, 30]. The allocated groups were Group 1 (G1): Vehicle controls received normal saline; Groups 2 (G2): ISO control rats were subcutaneously injected with ISO at a dose of 85 mg/kg body weight on days 19 and 20 (at an interval of 24 h) to induce myocardial infarction (MI) [31, 32]; Group 3 (G3): animals were orally pre-and co-administered with SCA at a dose of 5 mg/kg body weight for 20 days and subcutaneously with ISO on days 19 and 20; Group 4 (G4): animals were orally pre-and co-administered with SCA at a dose of 10 mg/kg body weight for 20 days and subcutaneously with ISO on days 19 and 20. These doses were selected based on previous research and established safe ranges within the literature [15, 24, 33]. On the 21st day of the experiment, the animals were euthanized using CO2 asphyxiation (Fig. 1A). During the euthanasia procedure, organ samples, specifically heart tissue were collected. Furthermore, whole blood samples were obtained for the isolation of serum. Additionally, organ and body weights were measured as part of the necropsy process.

### Determination of myocardial infarct size and heart weight/ body weight

After the necropsy, body weights were taken, hearts were excised out, rinsed in saline and weighed and heart-to-body weights were calculated. To assess the ISO-indued myocardial infarction in rats, myocardial infarct size was measured using 2,3,5-triphenyltetrazolium chloride (TTC) staining method [34, 35]. Briefly, the hearts were dissected and sliced into transverse sections of equal thickness. A 1% TTC solution was prepared in PBS or another buffer. The heart slices were incubated in the TTC solution at 37 °C for 20–30 min. Viable myocardial tissue metabolized TTC to a red formazan product, while infarcted tissue remained unstained, enabling the differentiation and quantification of the infarcted area. Following incubation, the heart slices were visually examined, and images were captured using an imaging system to differentiate between viable and infarcted tissue based on their staining characteristics.

### Biochemical analysis

On the day of termination, i.e., day 21, rats were kept overnight fasting with water allowed and euthanized by CO_2_ asphyxiation. Before sending animals for euthanization, blood was collected in tubes without anti-coagulant, left to coagulate at normal room conditions for not less than 30 min and centrifuged at 3000 rpm for 15 min to collect sera. The collected sera samples were then used to analyze the cardiac functional markers such as Creatine Kinase-Myoglobin Binding (CK-MB), Cardiac Troponin I (cTnI) and Brain natriuretic peptide (BNP) were measured as U/L, ng/mL and pg. mL, they were respectively, using the ELISA kit method.

### Histopathology

To analyze cardiac histopathological changes in controls as well as treatment groups, tissues fixed in 10% neutral formalin saline were processed for Hematoxylin and Eosin (H & E) staining by following the steps such as trimming, processing (with increasing concentration of alcohol), embedding (in paraffin wax), microtome sectioning (as 5 μm thick sections) and placing on adhesive slides. The quality check of slides was done by blindfolded by a pathologist Olympus phase contrast microscope (Tokyo, Japan). A histopathology peer review was also performed after the study pathologist completed the slide reading.

### Immunohistochemistry and immunofluorescence

For immunohistochemistry, sections were deparaffinized, rehydrated and blocked for peroxidase action by adding 0.3% hydrogen peroxide. Sections were incubated overnight at 4 °C with anti-Nrf2 anti-Keap1 primary antibodies. Sections were incubated for one hour with appropriate secondary antibodies and then with streptavidin peroxidase. Sections were then added with diaminobenzidine (DAB) for five minutes. Finally, sections were processed for staining with hematoxylin. Also, to ensure the specificity negative control was maintained with normal rabbit serum in place of the primary antibody.

For immunofluorescence, sections were incubated overnight at 4 °C with anti-NFKB and anti-P38 antibodies. Sections were then incubated for one hour with appropriate secondary antibodies conjugated with Dylight 594 (red). Sections were then stained with 4′,6-diamidino-2-phenylindole (DAPI), mounted with mountant, and observed under a confocal microscope. Meanwhile, specificity was ensured by maintaining negative control with normal rabbit serum in place of primary antibodies.

### Oxidative and anti-oxidative status in the heart

The cardiac levels of malondialdehyde (MDA), nitric oxide (NO), and glutathione (GSH) in nmol/mg protein, as well as enzyme activity levels of superoxide dismutase (SOD), catalase (CAT), and glutathione peroxidase (GPx) in U/mg protein, U/mg protein and GSH oxidized/min/mg of protein, respectively were determined using manufacturer’s protocol.

### Quantitative reverse transcription polymerase chain reaction (RT-PCR) analysis

RT-PCR was performed to quantify the PI3K, Akt, Nrf2, Keap1, NQO1, HO-1, Tlr4, NFKß-p65, IKKβ, ERK, JNK and P38 genes in cardiac tissue. Total RNA was extracted from the Thermo Fisher Scientific TM Fermentas RNA isolation kit based on the manufacturer’s instructions. RNA content was quantified with nanodrop and reverse transcribed for cDNA using RevertAid™ First Strand cDNA Synthesis kit (Thermo Fisher Scientific TM Fermentas). RT-PCR was performed using the SYBR Green PCT master mix kit provided by Thermo Fischer ScientificTM Invitrogen. The relative quantification of respective genes was done after normalizing with the housekeeping gene, i.e., β-actin. The primer sets used for the reactions are as follows.

**Table 1:** 

Nrf2	F 5’-CATTTGTAGATGACCATGAGTCGC-3’
	R 5’-ATCAGGGGTGGTGAAGACTG-3’
Keap 1	F 5’-CTTCGGGGAGGAGGAGTTCT-3’
	R 5’-CGTTCAGATCATCGCGGCTG-3’
Nqo1	F 5’-GACATCACAGGGGAGCCG-3’
	R 5’-CTCAGGCGGCCTTCCTTATAC-3’
Ho-1	F 5’-GTGCACATCCGTGCAGAGAA-3’
	R 5’-GTGCACATCCGTGCAGAGAA-3’
Tlr4	F 5’-AGTGTATCGGTGGTCAGTGTGCT-3’
	R 5’-AAACTCCAGCCACACATTCC-3’
NFKß-p65	F 5’-TCAGGAAGAGGTTTGGATGC-3’
	R 5’-AGCCCCTAATACACGCCTCT-3’
IKKß	F 5’-GCCTCTTCTCATTCCTGCTTG-3’
	R 5’-CTGATGAGAGGGAGGCCATT-3’
ERK	F 5’-CTTGGCATCCGCACTCTG-3’
	R 5’-CTGAAGCCTGGCAACCTG-3’
JNK	F 5’-TTCCATTGTGGGTAGGTGG-3’
	R 5’-CTTACAGCTTCCGCTTCAG-3’
P38	F 5’--CCAGATGCCGAAGATGAACT-3’
	R 5’-GGGCTGCTGTGATCCTCTTAT-3’
β-actin	F 5’ ?CACGATGGAGGGGCCGGACTCATC-3’
	R 5’-TAAAGACCTCTATGCCAACACAGT-3’

### Determination of levels of inflammatory markers in cardiac tissue of rats

Cardiac levels of NF-kB-p65, TLR4, TNF-α, IL-6 and IL-1β were determined with the help of respective ELISA kits and the analyses were done as per instructions given by the manufacturer.

### Statistical analysis

The current study demonstrated results as mean ± SEM of control and experimental groups. The statistical method of analysis to determine statistical significance (p < 0.05) between different groups was a one-way analysis of variance (ANOVA) followed by Tukey’s post hoc test in a validated statistical software SPSS version 21 (SPSS, Chicago, IL).

## Results

### Effects of SCA on Myocardial Infarct Size and heart/body weight in ISO-injected rats

The administration of ISO resulted in a significant increase (p < 0.05) in myocardial infarct size compared to the control group (Fig. 1B & D). Conversely, rats treated with SCA at doses of 5 and 10 mg/kg to ISO-treated rats, exhibited a significant reduction (p < 0.05) in myocardial infarct size compared to ISO-treated rats (Fig. 1B & D). Furthermore, the 10 mg/kg dose of SCA showed a significantly greater decrease in myocardial infarct size compared to the 5 mg/kg dose.

A significant increase in the heart/body weight ratio, indicating heart enlargement, was observed in ISO-injected rats compared to control rats. However, pre- and co-treatment of ISO-injected animals with SCA at 5 and 10 mg/kg resulted in a significant (p < 0.05) reduction in the heart/body weight ratio compared to only ISO-injected rats, suggesting the cardioprotective effects of SCA in ISO-induced rats (Fig. 1C). There was no significant difference between SCA treatment at 5 and 10 mg/kg for heart/body weight ratio. Additionally, the high dose of SCA (10 mg/kg body weight) normalized the heart/body weight ratio in ISO-administered rats.

### Effects of SCA on cardiac functional markers in ISO-injected rats

**Fig. 2:**
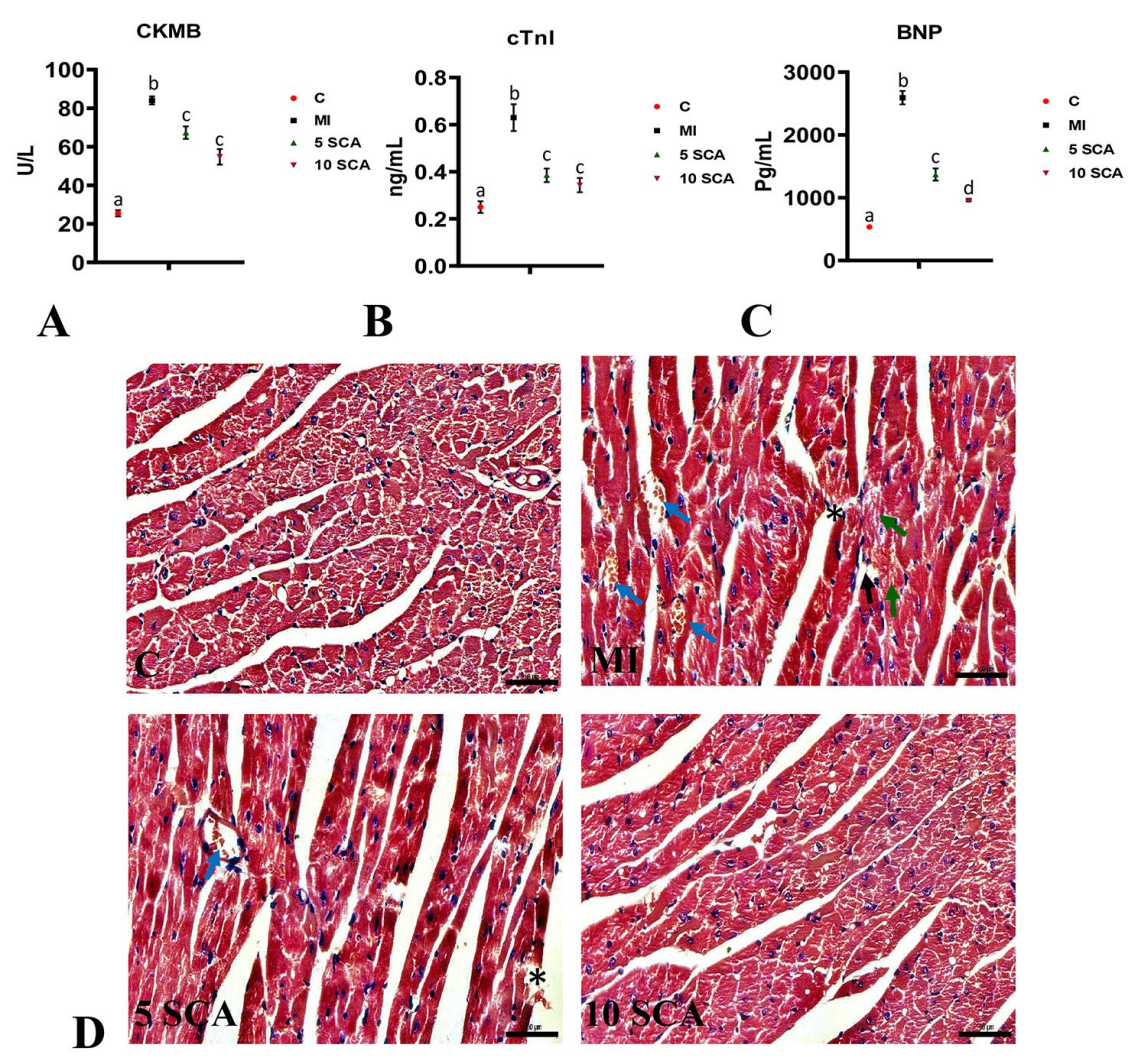
Effect of SCA treatment on ISO-induced changes in cardiac biochemical markers in rats. **(A)**: Creatine kinase-MB (CK-MB); **(B)**: Cardiac Troponin I (cTnI); **(C)**: Brain natriuretic peptide (BNP); **(D)** Histopathological changes through hematoxylin and eosin (H&E) staining in heart. ISO or SCA treated group shows myocardial damage with fragmented and separated cardiac muscle fibers (black arrow). Vascular leakage of RBCs is indicated by blue arrows. The ISO group also exhibits necrosis (*) and inflammatory cell infiltration. Fibrosis is shown by the green arrow. C: Control rats; MI: Isoproterenol (ISO) induced myocardial infarction (MI) in rats; 5 SCA: Treatment of ISO-induced rats with 5 mg SCA/ kg body weight; 10 SCA: Treatment of ISO-induced rats with 10 mg SCA/ kg body weight. The data is presented as mean ± SD (n = 10). Statistical analysis was performed using One-Way ANOVA followed by Tukey’s post hoc analysis to compare differences between groups. Mean values with different superscripts indicate significant differences at a significance level of p < 0.05. Scale bar = 50 μm. Magnification: X400

In this study, significant alterations in cardiac functionality biomarkers were observed. Serum levels of CK-MB (Fig. 2A), cTnI (Fig. 2B), and BNP (Fig. 2C) showed a significant (p < 0.05) increase in ISO-treated rats compared to control rats. However, treatment with SCA at 5 or 10 mg/kg body weight in ISO-injected rats resulted in a significant (p < 0.05) reduction in serum levels of CK-MB, cTnI, and BNP compared to ISO-only rats. Additionally, there was no significant difference between the 5 or 10 mg/kg body weight in terms of CK-MB and cTnI, but the 10 mg/kg body weight significantly decreased BNP compared to the 5 mg/kg body weight.

### Effect of SCA on Histopathology in the heart of ISO-treated myocardial infracted mice

Histopathological examination of the H&E-stained slides (Fig. 2D) revealed clear distinctions between the groups. The heart sections from the control rats displayed a normal architecture with intact cardiac muscle fibers, characterized by well-defined striations and intact cell membranes. In contrast, the heart sections from the ISO-induced rats exhibited pronounced myocardial damage, including distorted cardiomyocytes, disrupted myocardial fibers, and areas of necrosis. The ISO group also showed vascular changes, fragmentation, focal cell infiltration, patchy necrotic areas, and hyalinization of muscle fibers. In addition, increased levels of inflammation and fibrosis were observed in the ISO group. However, the heart sections from the SCA and ISO-treated group demonstrated a significant protective effect against these histopathological alterations. These sections exhibited reduced necrosis, decreased interstitial space, preserved architectural integrity, and less myocardial damage compared to the ISO group.

### Effect of SCA on Oxidative and anti-oxidative status in the heart of ISO-treated myocardial infracted mice

**Fig. 3:**
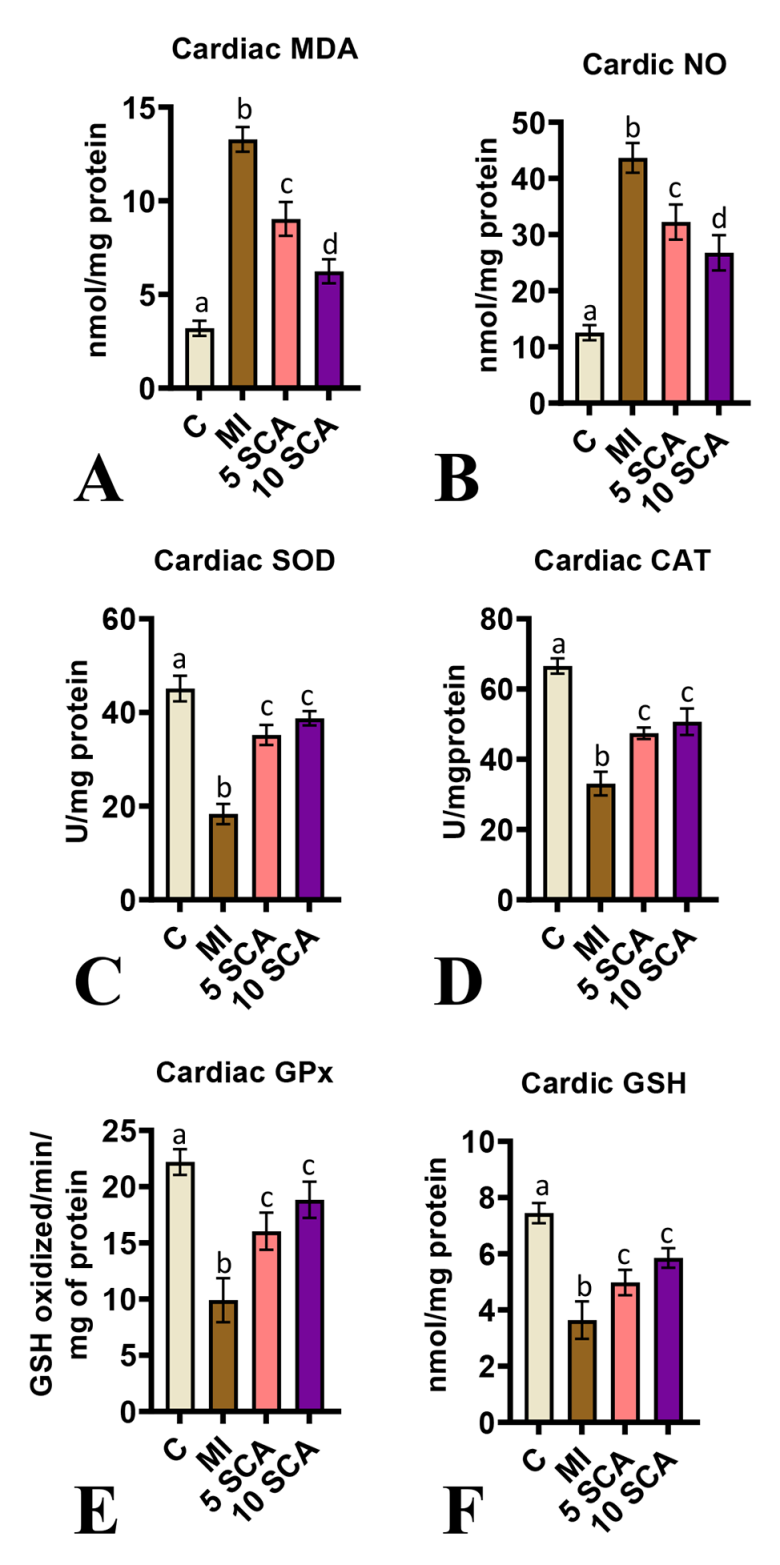
Effect of SCA treatment on ISO-induced changes in rats’ cardiac oxidative stress and antioxidant defense markers. **(A)**: Malondialdehyde (MDA); **(B)**: Nitric oxide (NO) **(C)**: superoxide dismutase (SOD); **(D)**: Catalase (CAT); **(E)**: Glutathione peroxidase (GPx); **(F)**: Glutathione (GSH). C: Control rats; MI: Isoproterenol (ISO) induced myocardial infarction (MI) in rats; 5 SCA: Treatment of ISO-induced rats with 5 mg SCA/ kg body weight; 10 SCA: Treatment of ISO-induced rats with 10 mg SCA/ kg body weight. The data is presented as mean ± SD (n = 10). Statistical analysis was performed using One-Way ANOVA followed by Tukey’s post hoc analysis to compare differences between groups. Mean values with different superscripts indicate significant differences at a significance level of p < 0.05

A comprehensive analysis of biochemical, molecular, and immunochemical biomarkers was conducted to assess the oxidative and anti-oxidative status of cardiac tissue. ISO-treated rats exhibited severe oxidative stress, characterized by a significant (p < 0.05) increase in MDA levels (Fig. 3A) and NO levels (Fig. 3B), along with a significant decrease (p < 0.05) in GSH levels (Fig. 3F) and enzyme activities of SOD (Fig. 3C), CAT (Fig. 3D), and GPx (Fig. 3E) in the heart compared to control rats. However, pre- and co-treatment with SCA at 5 or 10 mg/kg body weight significantly protected against these oxidative alterations induced by ISO, leading to a significant reduction in MDA and NO levels and a significant increase in GSH levels, as well as SOD, CAT, and GPx activities, compared to ISO-only rats. Furthermore, the 10 mg/kg dose of SCA exhibited a significantly greater decrease in MDA and NO levels compared to the 5 mg/kg dose, while there was no significant difference in GSH levels and antioxidant enzyme activities between the 5 and 10 mg/kg doses.

**Fig. 4:**
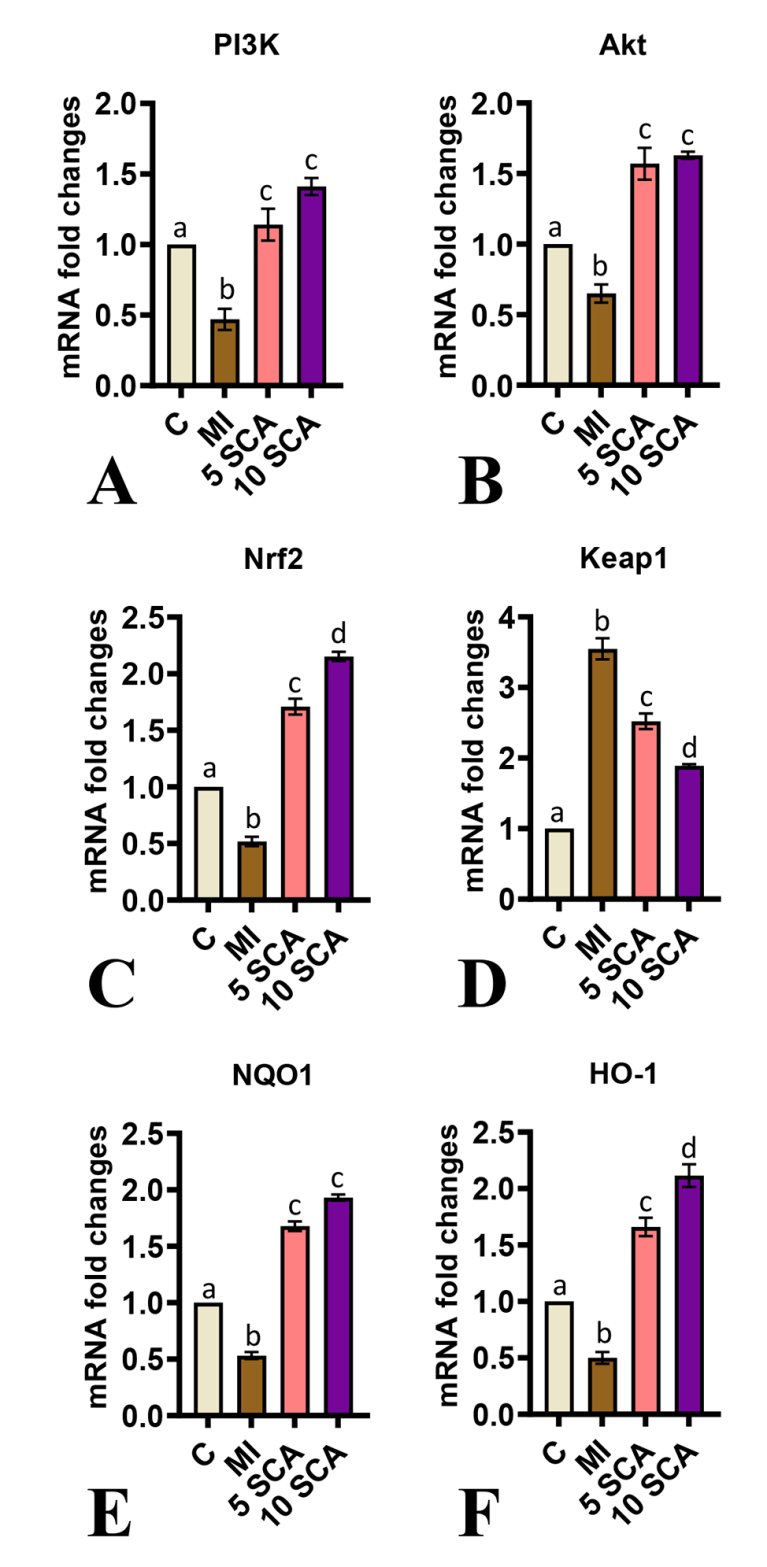
Effect of SCA treatment on ISO-induced changes in PI3K/Akt/Nrf2/ARE signaling in cardiac tissue of rats. Relative mRNA expression of **(A)**: *PI3K*; **(B)**: *Akt*; **(C)**: *Nrf2*; **(D)**: *Keap1*; **(E)**: *Nqo1;***(F)**: *Ho-1*. C: Control rats; MI: Isoproterenol (ISO) induced myocardial infarction (MI) in rats; 5 SCA: Treatment of ISO-induced rats with 5 mg SCA/ kg body weight; 10 SCA: Treatment of ISO-induced rats with 10 mg SCA/ kg body weight. The data is presented as mean ± SD (n = 10). Statistical analysis was performed using One-Way ANOVA followed by Tukey’s post hoc analysis to compare differences between groups. Mean values with different superscripts indicate significant differences at a significance level of p < 0.05

In addition to biochemical parameters, molecular analyses were conducted to assess gene expressions of PI3K (Fig. 4A), Akt (Fig. 4B), Nrf2 (Fig. 4C), Keap1 (Fig. 4D), Nqo-1 (Fig. 4E), and Ho-1 (Fig. 4F). The results revealed a significant downregulation in mRNA expressions of PI3K, Akt, Nrf2, Nqo-1, and Ho-1, as well as an upregulated mRNA expression of Keap1 in the cardiac tissue of ISO-induced rats compared to control rats. Conversely, pre- and co-treatment of ISO-injected rats with 5 or 10 mg/kg body weight of SCA showed significant upregulation in gene expressions of PI3K, Akt, Nrf2, Nqo-1, and Ho-1, as well as significant downregulation in Keap1 gene expression in cardiac tissue compared to rats treated with ISO alone. However, there were significant differences in Nrf2 and Ho-1 mRNA levels between the 10 mg/kg and 5 mg/kg body weight doses of SCA.

**Fig. 5:**
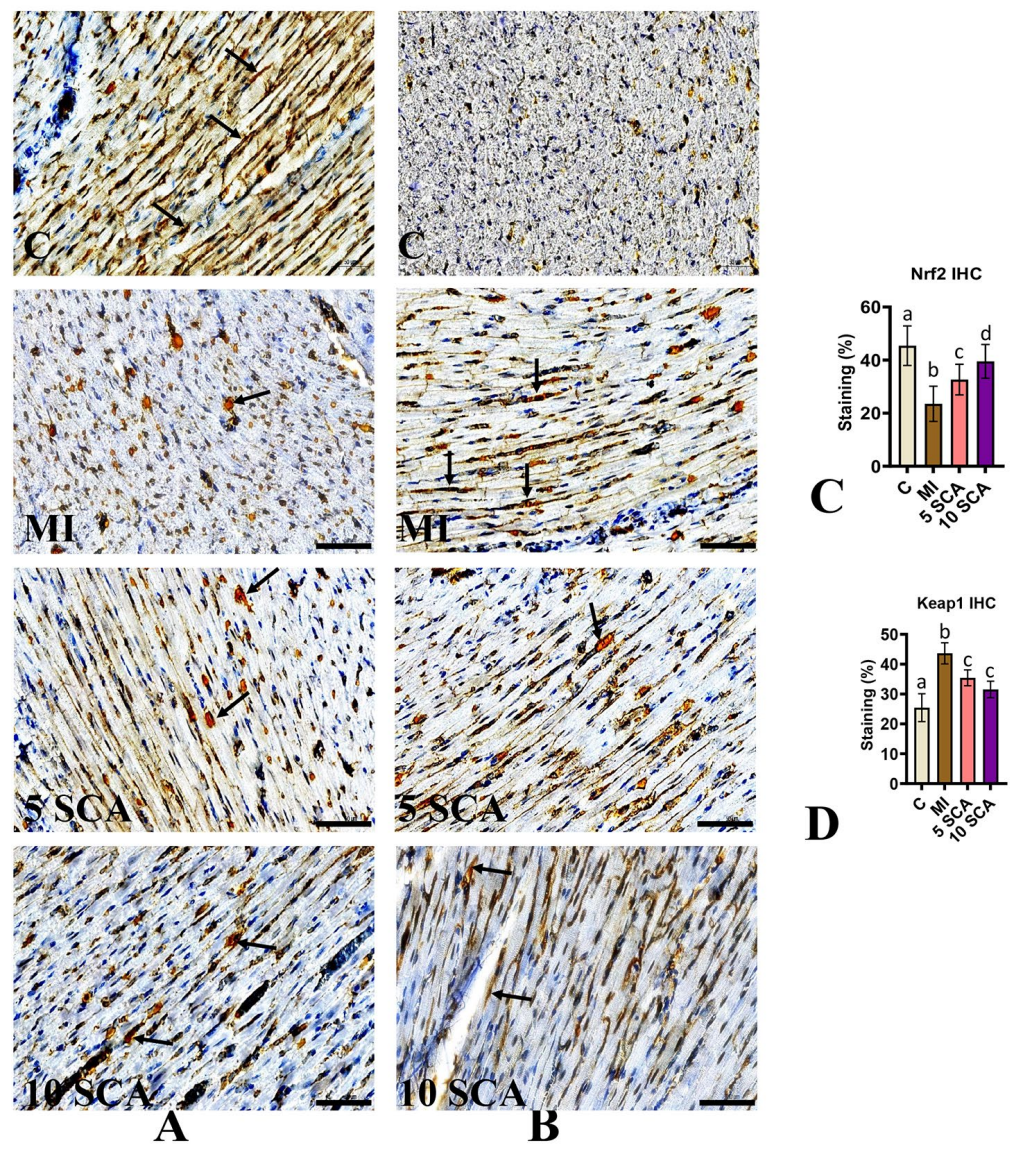
Immunohistochemical staining for myocardial (**A**) Nrf2 and (**B**) Keap 1 distribution. (**C**) Nrf2 and (**D**) Keap 1 brown staining indicate the cells with positive staining. C: Control rats; MI: Isoproterenol (ISO) induced myocardial infarction (MI) in rats; 5 SCA: Treatment of ISO-induced rats with 5 mg SCA/ kg body weight; 10 SCA: Treatment of ISO-induced rats with 10 mg SCA/ kg body weight. The data is presented as mean ± SD (n = 10). Statistical analysis was performed using One-Way ANOVA followed by Tukey’s post hoc analysis to compare differences between groups. Mean values with different superscripts indicate significant differences at a significance level of p < 0.05. The black arrow indicates the protein expression specifically in cardiomyocytes. (Scale bar = 50 μm; magnification, x400)

The study further investigated the immuno-histochemical staining of Nrf2 and Keap1 on cardiac sections of rats. The findings supported the molecular analyses, revealing decreased protein distribution of Nrf2 and increased protein distribution of Keap1 in cardiac tissue of ISO-administered rats. Conversely, SCA treatment at doses of 5 or 10 mg/kg body weight resulted in elevated protein distribution of Nrf2 and reduced protein distribution of Keap1 in cardiac tissue of ISO-induced rats (Fig. 5A and B). The percentage staining of Nrf2 and Keap1 is presented in Fig. 5C and D, respectively. However, significant differences in Nrf2 protein distribution were observed between the 10 mg/kg and 5 mg/kg body weight doses of SCA.

### Effect of SCA on inflammatory signaling pathways and markers in cardiac tissue of ISO-treated rats

**Fig. 6:**
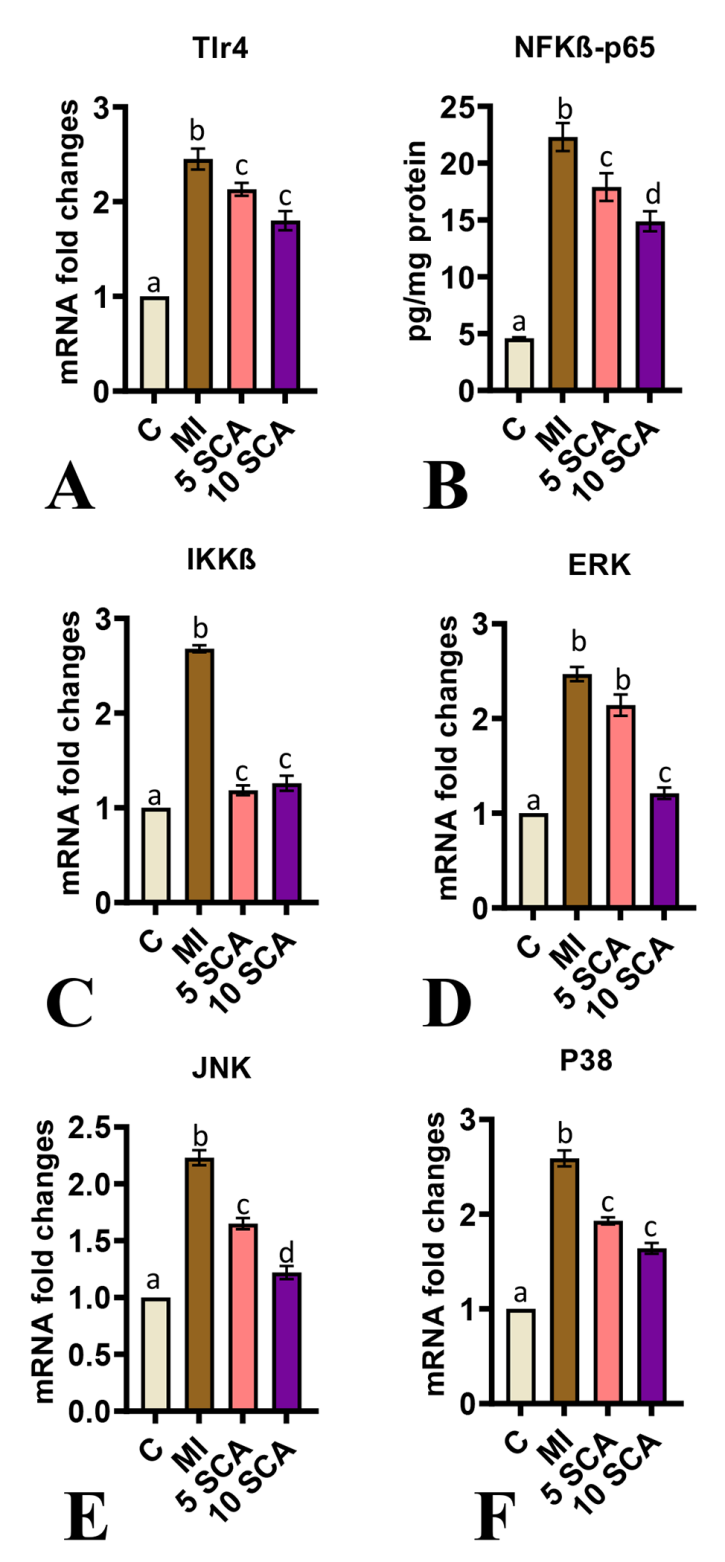
Effect of SCA treatment on ISO-induced changes in TLR4/MAPK/NF-kB signaling in cardiac tissue of rats. Relative mRNA expression of **(A)**: *Tlr4*; **(C)**: *IKKβ;***(D)**: *ERK*; **(E)**: JNK; **(F)**: *P38;* ELISA reading for **(B)** NF-kB-p65 levels; C: Control rats; MI: Isoproterenol (ISO) induced myocardial infarction (MI) in rats; 5 SCA: Treatment of ISO-induced rats with 5 mg SCA/kg body weight; 10 SCA: Treatment of ISO-induced rats with 10 mg SCA/ kg body weight. Data are Mean ± SEM (n = 10). *p < 0.05 versus control, and ^*p* < 0.05 versus ISO. The data is presented as mean ± SD (n = 10). Statistical analysis was performed using One-Way ANOVA followed by Tukey’s post hoc analysis to compare differences between groups. Mean values with different superscripts indicate significant differences at a significance level of p < 0.05

The levels of NF-kB-p65 (Fig. 6B) and mRNA expressions of Tlr4 (Fig. 6A), IKKβ (Fig. 6C), ERK (Fig. 6D), JNK (Fig. 6E), and P38 (Fig. 6F) were evaluated in the cardiac tissue of rats. The results showed significantly elevated levels of NF-kB-p65, along with upregulated gene expressions of Tlr4, IKKβ, ERK, JNK, and P38 MAPK in the cardiac tissue of the ISO-administered rats compared to control rats. In contrast, SCA treatment in ISO-induced rats led to significantly reduced levels of NF-kB-p65, as well as downregulated mRNA expressions of Tlr4, IKKβ, ERK, JNK, and P38 MAPK, compared to the levels observed in the cardiac tissue of ISO-only treated rats. Furthermore, there was a significant decrease in ERK mRNA levels specifically in the 10 mg/kg body weight group, not in the 5 mg/kg body weight group.

**Fig. 7:**
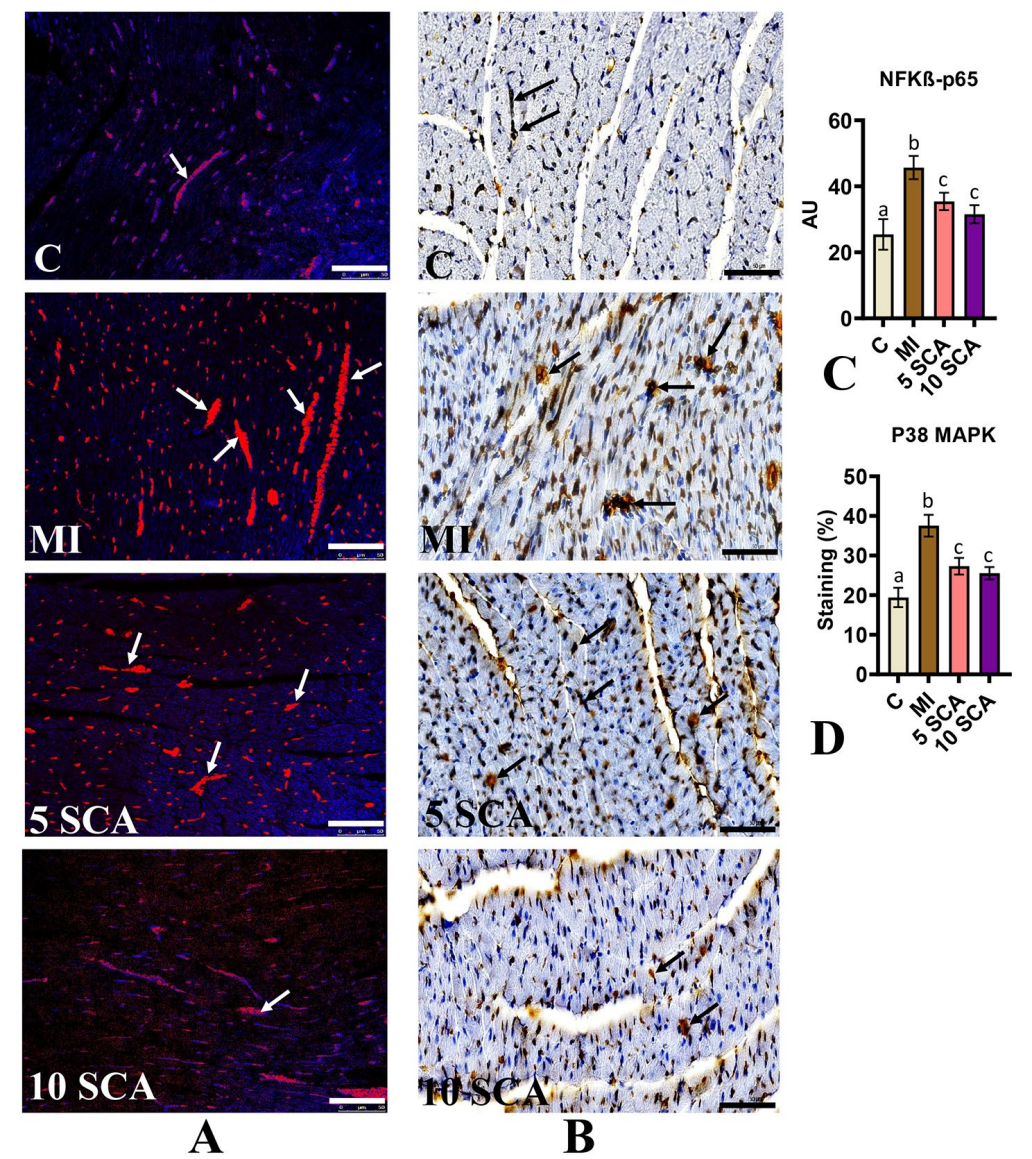
Immunofluorescent results of myocardial (**A**) NF-κΒ p65 and immunohistochemical staining (**B**) P38 MAPK; (**C**) Fluorescence intensity (red color) of NF-κΒ p65 and (**D**) p38 MAPK (% of brown staining). C: Control rats; MI: Isoproterenol (ISO) induced myocardial infarction (MI) in rats; 5 SCA: Treatment of ISO-induced rats with 5 mg SCA/ kg body weight; 10 SCA: Treatment of ISO-induced rats with 10 mg SCA/ kg body weight. The data is presented as mean ± SD (n = 10). Statistical analysis was performed using One-Way ANOVA followed by Tukey’s post hoc analysis to compare differences between groups. Mean values with different superscripts indicate significant differences at a significance level of p < 0.05. The black and white arrow indicates the protein expression specifically in cardiomyocytes. (Scale bar = 50 μm; magnification, x400)

Additionally, the findings were supported by immunofluorescence staining of NFKB and P38 proteins. Cardiac sections of ISO-treated rats exhibited a significant increase in protein expression of NF-kB-p65 (Fig. 7A) and P38 MAPK (Fig. 7B) compared to control rats. However, these expressions were significantly reduced in SCA + ISO-treated rats compared to ISO-only rats. The fluorescence intensity of NF-kB-p65 proteins is shown in arbitrary units (AU) (Fig. 7C), and the percentage staining of P38 MAPK is depicted in Fig. 7D. There was no significant difference between the 5 and 10 mg/kg body weight groups in SCA treatment of ISO-induced rats.

**Fig. 8:**
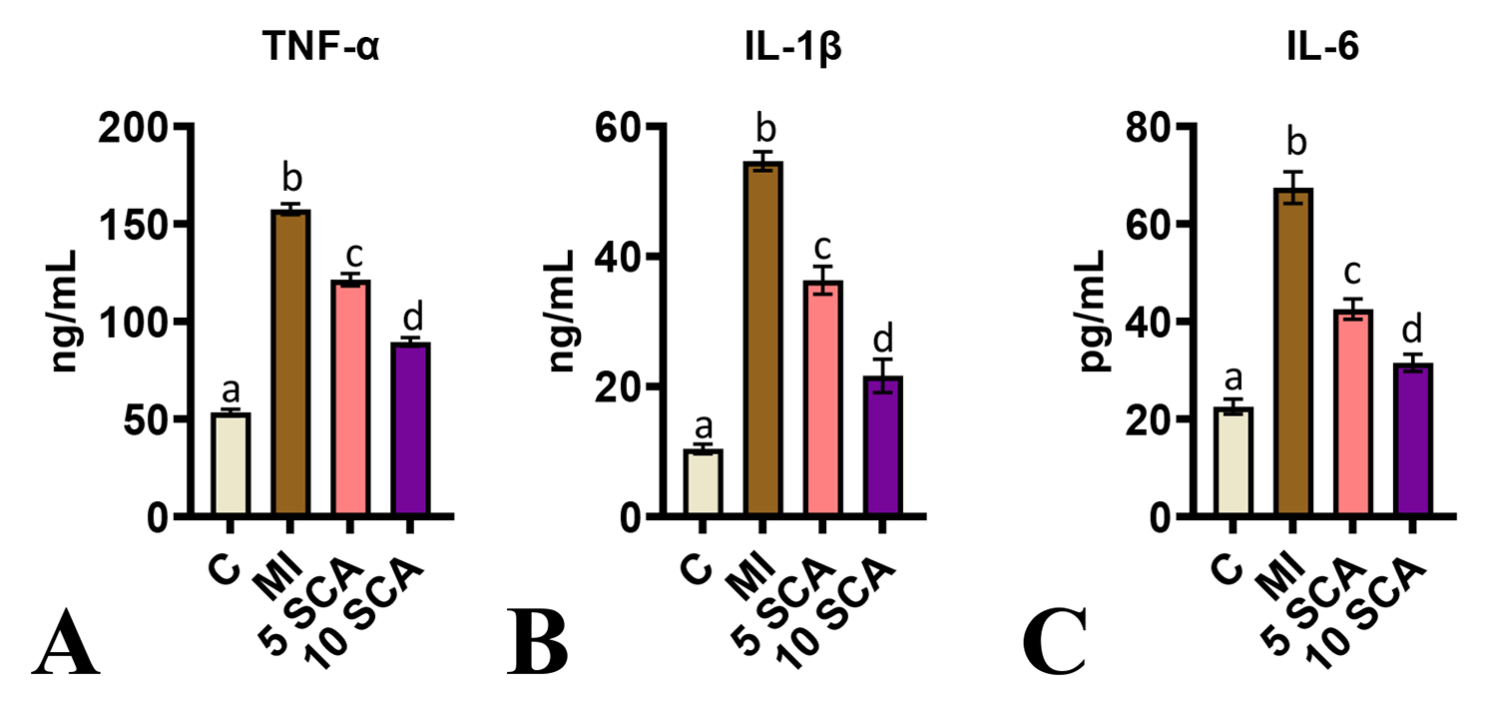
ELISA assay for cardiac (**A**) TNF-α, (**B**) IL-1β, (**C**) IL-6; C: Control rats; MI: Isoproterenol (ISO) induced myocardial infarction (MI) in rats; 5 SCA: Treatment of ISO-induced rats with 5 mg SCA/ kg body weight; 10 SCA: Treatment of ISO-induced rats with 10 mg SCA/kg body weight. The data is presented as mean ± SD (n = 10). Statistical analysis was performed using One-Way ANOVA followed by Tukey’s post hoc analysis to compare differences between groups. Mean values with different superscripts indicate significant differences at a significance level of p < 0.05

Furthermore, the levels of TNF-α (Fig. 8A), IL-1β (Fig. 8B), and IL-6 (Fig. 8C) were significantly elevated in ISO-treated rats compared to control rats. However, SCA administration significantly reduced the increase in TNF-α, IL-6, and IL-1β in ISO-treated rats. There was a significant difference in TNF-α, IL-6, and IL-1β levels between the 5 and 10 mg/kg body weight groups in SCA treatment of ISO-induced rats.

## Discussion

Recently, CVD particularly MI-mediated mortality or morbidity has been of great concern and increasing at an alarming rate. SCA, a natural derivative from the fruits of *S. chinensis* is well-known for its health-promoting effects. The present study examined the possible protective effects of SCA against ISO-induced cardiac injury. The results revealed that SCA had shown significant cardio-protective potential by ameliorating myocardial infarct size, cardiac functional markers, oxidative stress, inflammation markers and histopathological alterations in the heart of rats. Furthermore, the findings also showed that SCA protects ISO-induced oxidative stress and inflammation by activating PI3K-AKT/Nrf2/ARE pathway and suppressing TLR4/MAPK/NF-κB pathways, respectively.

Acute myocardial infarction is induced in rats by two subcutaneous injections of ISO at 24 h. Historically, the ISO-induced MI, a non-selective β-agonistic approach is the best reliable model to mimic human AMI due to its simple, non-invasive, reproducible, and lower mortality rate. Recently, A Elshaer, AE Sobhy, MM Elalfy, AM Ghoneem, AK Elhawary and MA Attia [36] proved that of three experimental approaches in rats, i.e., surgical approach, two subcutaneous injections of ISO a day apart or intraperitoneal injections of Doxorubicin, induction of AMI by ISO shows the highest level of reliability in simulation to AMI in humans. The current study supports previous scientific evidence by demonstrating a significant increase in myocardial infarct size, heart/body weight ratio, and intensification of various serum levels of cardiac markers such as CK-MB, cTnI, and BNP in the ISO-induced MI model [5, 6, 37]. Evaluating these cardiac markers has been considered a gold standard approach to assessing cardiac functionality. Any loss of functional integrity and membrane permeability leads to the leakage of markers into the bloodstream [38, 39]. However, pre- and co-treatment of ISO-administered rats with SCA at 05 or 10 mg/kg body weight for 20 consecutive days resulted in a significant decrease in myocardial infarct size, heart-to-body weight ratio and serum levels CK-MB, cTnI and BNP as an indication of cardioprotective effect of SCA. These results were further supported by significant protection from cardiac pathological changes observed in this study. Supporting this, earlier R Chang, Y Li, X Yang, Y Yue, L Dou, Y Wang, W Zhang and X Li [27] showed that SCA could protect myocardial ischemia–reperfusion (I/R) injury by influencing cardiac function and myocardial infarct size.

A plethora of scientific evidence has demonstrated that the oxidative homeostasis disequilibrium in myocardial cells instigates excessive formation of cytotoxic free radicals under MI conditions [40, 41]. If enough antioxidants do not counteract the surplus production of free radicals, then free radicals can interact with cellular lipoproteins and form lipid peroxides, which can have disastrous effects on cardiac cells [42, 43]. In this study, loss of cellular oxidative equilibrium was proven by significantly high levels of MDA and NO with significantly lower levels of GSH and activities of SOD, CAT and GPx in cardiac tissue. The results are corroborated by previous findings [5, 6]. Conversely, SCA treatment in ISO-induced rats prevented a rise in oxidative stress markers and a fall in antioxidant defense markers exemplifying the antioxidant potential of SCA. Earlier, it was demonstrated that *S. chinensis* efficiently improves liver function in patients by significantly increasing antioxidant capacity and decreasing oxidative active substances [44, 45]. Furthermore, the non-clinical study revealed that SCA conspicuously improves cognitive deficits, which might be due to its antioxidant potential through restoring activities of SOD, GPx, GSH and MDA [46].

To know the molecular mechanisms behind the antioxidant nature of SCA, the critical regulatory pathway involved in the protection of oxidative stress, i.e., the Nrf2 signaling pathway in cardiac cells of rats is studied. Nrf2 is an essential transcription factor that regulates cytoprotective and antioxidant genes and enzymes. Studying this pathway helps pave the molecular way to protect against cardiac oxidative damage. Generally, Nrf2 is bound by its cytosolic negative regulator, Keap1, which hinders its entry into the nucleus. In a stimulated state, Nrf2 evades from Keap1. It translocate to the nucleus and binds to the antioxidant response element (ARE) to activate various genes involved in antioxidant defenses to protect cells from oxidative stress. Furthermore, the critical role of Nrf2 in antioxidant activity was evidenced by its inhibition of Nrf2 by its specific inhibitor, weakening its antioxidant ability [47]. In the present study, a deranged Nrf2 signaling cascade was evident through reduced expression of Nrf2 (gene and protein), Nqo-1 (gene) and Ho-1 (gene) and increased gene and protein expression of Keap1 in an ISO-induced rat model. These observations align with previous studies showing ISO-mediated derangement in cardiac cellular Nrf2 signaling pathways. On the other hand, SCA treatment activated Nrf2 signaling through increased expression of Nrf2 and reduced expression of Keap1, thereby Nrf2 transactivates genes such as Nqo-1 and Ho-1 to confer antioxidant defense against oxidative stress. Supporting this, H Lin, X Zhang, J Liu, L Yuan, J Liu, C Wang, J Sun, J Chen, S Jing and H Li [13] reported that SCA improves learning and memory skills by modulating the Nrf2/Keap1/ARE pathway in mice.

The phosphatidylinositol 3-kinase (PI3K)/Akt pathway has also been considered pivotal in modulating cellular defense as an upstream regulator to Nrf2-mediated antioxidant response [47–49]. PI3K/Akt suppression with its specific inhibitor could modulate cellular defense by inhibiting phosphorylation of Akt and subsequent activation of Nrf2/HO-1 expression [47, 50]. This study’s results have shown a significant reduction in PI3K and Akt mRNA expression in ISO-induced rat heart tissues compared to respective gene expressions in control rats. Contrary to this, SCA administration of SCA at 5 or 10 mg/kg body weight to ISO-challenged rats has resulted in significant upregulation in PI3K and Akt gene expression compared to the same gene expressions in only ISO-treated rats. As supportive evidence, earlier pre-treatment of human renal tubular epithelial cells i.e., HK-2 cells with SCA showed significant protection against H/R injury through activation of the PI3K/Akt pathway [17].

In addition to oxidative stress, inflammation has significantly contributed to MI-induced cardiac injury [51, 52]. Many preclinical studies have demonstrated that DAMPs produced because of myocardial injury stimulate the TLR4 signaling pathway. Also, previous nonclinical studies have proved that TLR4 initiates inflammatory responses via the activation of NF-kB and Mitogen-activated protein kinases (MAPKs) signaling cascades [53, 54]. In an unstimulated stimulation, NF-kB is under the custody of IkB in the cytoplasm, however, in stressed situations inhibitor-kB (IkB) kinase (IKKB) degrades IkB and thereby NF-kB translocated to the nucleus to activate various inflammatory mediators such as TNF-α, IL-1β and IL-6 [55]. The findings of this study showed upregulated gene expressions of Tlr4, NF-κB p65, IKKβ, ERK, JNK and p38 and significantly higher levels of TNF-α, IL-1β and IL-6 in cardiac tissue of ISO-induced rats. Furthermore, the immunofluorescence studies supported gene expression results by showing increased protein expressions of NFKB and p38 in the cardiac sections of ISO-treated rats. The observed results were well supported by various other non-clinical studies in ISO-induced animals [25, 56, 57]. Conversely, the alterations observed in various inflammatory markers were markedly attenuated by pre- and co-treatment of ISO-treated rats with SCA at either 5 or 10 mg/kg body weights. These findings indicate the anti-inflammatory role of SCA by suppressing inflammation by inhibiting TLR44/MAPK/NF-kB signaling and its subsequent production of inflammatory cytokines. H Wang, J Che, K Cui, W Zhuang, H Li, J Sun, J Chen and C Wang [15] in vivo and in vitro study results showing significant protection against liver fibrosis through suppression of inflammatory factors and different signaling pathways including MAPK and NF-kB cascades support the present observations. Furthermore, SCA prevented in vivo and in vitro LPS-induced activation of NF-kB and a rise in different inflammatory cytokines expression [58].

## Conclusion

In conclusion, the pre- and co-treatment of ISO-treated rats with SCA at doses of 5 or 10 mg/kg body weight for 20 days demonstrated significant protection against cardiac injury in the MI rat model. SCA exhibited beneficial effects on various parameters including heart/body weight ratio, myocardial infarct size, cardiac biomarkers, oxidative stress, inflammation, and histopathological changes in cardiac tissue. The study also revealed that SCA exerted its protective effects by activating the PI3K-AKT/Nrf2/ARE pathway and suppressing the TLR4/MAPK/NF-κB pathways. These findings highlight the potential medicinal and pharmacological properties of SCA and its relevance to current myocardial infarction treatment strategies. However, further verification in clinical scenarios is necessary. Limitations include the limited generalizability of the ISO-induced model to human myocardial infarction and the unclear optimal dose and treatment duration in humans. Recommendations include conducting clinical trials, comparative studies, assessing long-term effects and safety profiles, optimizing formulations, and addressing study limitations to advance the clinical application of SCA.

.

## Data Availability

The datasets used and/or analyzed during the current study available from the corresponding author on reasonable request.

## Abbreviations

ACE: Angiotensin-Converting Enzyme
Akt: Protein kinase B
ARE: Antioxidant response element
BNP: Brain natriuretic peptide
C5AR1: Complement component 5a receptor 1
CAT: Catalase
CK-MB: Creatine Kinase-Myoglobin Binding
cTnI: Cardiac Troponin I
CVDs: Cardiovascular diseases
DAB: Diaminobenzidine
DAPI: 4′,6-diamidino-2-phenylindole
ERK: Extracellular signal-regulated kinase
GPx: Glutathione peroxidase
GSH: Glutathione
HO-1: Heme oxygenase-1
IKKβ: Inhibitor of nuclear factor kappa-B kinase subunit beta
ISO: Isoproterenol
JNK: Jun N-terminal kinase
Keap1: Kelch-like ECH-associated protein 1
MDA: Malondialdehyde
MI: Myocardial infarction
NFKß: Nuclear factor kappa-light-chain-enhancer of activated B cells
NO: Nitric oxide
NQO1: NAD(P)H:quinone acceptor oxidoreductase
Nrf2: Nuclear factor erythroid 2-related factor 2
P38 MAPK: p38 mitogen-activated protein kinases
PI3K: Phosphatidylinositol-3 kinase
ROS: Reactive oxygen species
SCA: Schisantherin A
SOD: Superoxide dismutase
TLR4: Toll-like receptor 4
TTC: 2,3,5-triphenyltetrazolium chloride
